# The resveratrol monomer enhances resistance to bacterial pathogen through the activation of SA-NPR1 signaling pathway in *Arabidopsis*

**DOI:** 10.3389/fpls.2026.1834312

**Published:** 2026-09-28

**Authors:** Yuqiao Ma, Sun Ho Kim, Minh Le Anh Pham, Jungwoo Lee, Christian Alin Tonapa Sangka, Kyun Oh Lee, Jaesung Kim, Woo Sik Chung

**Affiliations:** Division of Applied Life Science (BK21 Four Program) and Plant Molecular Biology and Biotechnology Research Center, Gyeongsang National University, Jinju, Republic of Korea

**Keywords:** NPR1, pathogen resistance, phytoalexin, resveratrol, ROS, salicylic acid

## Abstract

Resveratrol is stilbene phytoalexin that exhibits antimicrobial activity in diverse plant species. Although its direct antimicrobial effects have been investigated extensively, its role and regulatory mechanism in plant immune signaling remain unclear. In this study, we demonstrate that resveratrol enhances resistance to bacterial pathogens through activation of endogenous defense pathways in *Arabidopsis thaliana*. Resveratrol treatment reduced disease symptoms and bacterial growth caused by *Pseudomonas syringae* pv. *tomato* DC3000 (*Pst* DC3000) without exerting direct antibacterial activity and increased transcription of *PATHOGENESIS-RELATED* (*PR*) genes. Resveratrol promoted translocation of NONEXPRESSOR OF PATHOGENESIS-RELATED GENES 1 (NPR1) into the nuclear and failed to confer resistance in the *npr1–1* mutant, which demonstrates that NPR1-mediated signaling is required for this response. Furthermore, resveratrol increased endogenous SA levels through transcriptional activation of SA biosynthesis genes. Accordingly, resveratrol-induced resistance and *PR1* expression were abolished in the SA-deficient plants, which indicates that resveratrol-induces pathogen resistance depends on the SA signaling. In addition, resveratrol triggered rapid accumulation of reactive oxygen species (ROS), suggesting that ROS may contributes to this immune response. Collectively, these findings demonstrate that resveratrol functions as an immune-activating molecule that enhances bacterial resistance through NPR1-dependent SA signaling and ROS accumulation in *Arabidopsis*.

## Introduction

Plants possess an intrinsic capacity to defend against pathogen invasion through the biosynthesis of phytoalexins, a class of antimicrobial secondary metabolites that are rapidly synthesized *de novo* in response to stress. These compounds restrict pathogen growth and spread and therefore constitute a critical component of plant immunity (Chan, 2002; Gambini et al., 2015; Li et al., 2016). Among diverse phytoalexins, resveratrol is a stilbene-type phytoalexin widely produced in various plant species, such as grapevine, peanut, and berry crops (Gambini et al., 2015; Duke, 2022). Similar to other stilbenes, resveratrol biosynthesis is strongly induced by various environmental stresses, including pathogen infection, ultraviolet irradiation, ozone exposure, and mechanical wounding (Jeandet et al., 1995; Ignatowicz and Baer-Dubowska, 2001; He and Yan, 2013; Pecyna et al., 2020). Resveratrol exhibits broad-spectrum antimicrobial activity against diverse phytopathogens. For example, resveratrol is accumulated significantly at infection sites in common bean following bacterial challenge, which supports its antibacterial function *in planta* (Cooper et al., 2022; Cooper, 2023). In addition, several studies have demonstrated that resveratrol inhibits the proliferation of diverse bacterial species *in vitro* (Chan, 2002; Cooper et al., 2022). These findings have established resveratrol as a direct antimicrobial compound.

Recent studies have expanded the functional scope of stilbenes, particularly resveratrol oligomers. Resveratrol-derived oligomers, such as hopeaphenol, suppress bacterial virulence through the repression of motility and the downregulation of genes associated with the type III secretion system (T3SS) or type III effector (T3Es) in *Pseudomonas syringae* pv. *tomato* DC3000 (*Pst* DC3000) (Kang et al., 2020a; Kang et al., 2022). These oligomeric compounds also enhance early immune responses, including the accumulation of reactive oxygen species (ROS) and the activation of pathogen-triggered cell death (Kang et al., 2022), which suggests dual anti-virulence and immune-priming activities. Although these findings identify resveratrol oligomers as biologically active regulators of plant-pathogen interactions, their precise molecular mechanism has not yet been elucidated.

In contrast to oligomeric stilbenes, the biological function of the resveratrol monomer in plant pathogen resistance remains largely undefined. Despite its well-established antimicrobial properties (Vestergaard and Ingmer, 2019; Cooper et al., 2022), it is unclear whether resveratrol enhances host resistance through modulation of endogenous defense signaling pathways. Importantly, previous reports have indicated that resveratrol monomers are likely to exhibit weaker antifungal activity than oligomeric derivatives (Kang et al., 2020b; Kang et al., 2022), which further obscures their potential contribution to plant pathogen resistance. Thus, whether resveratrol monomer functions as an immune-activating molecule, rather than solely as a chemical barrier to pathogens, remains an unresolved question.

Salicylic acid (SA) functions as key signaling molecule in plant defense and coordinates multiple immune responses, including pattern-triggered immunity (PTI), effector-triggered immunity (ETI), and systemic acquired resistance (SAR) (van Butselaar and Van den Ackerveken, 2020; Zhou and Zhang, 2020; Han et al., 2022; Kim et al., 2022; Kim and Lim, 2023). The SA signaling pathway largely depends on NONEXPRESSOR OF PATHOGENESIS-RELATED GENES 1 (NPR1) in *Arabidopsis* (Liu et al., 2005; Backer et al., 2019; Ding and Ding, 2020). Under normal conditions, NPR1 mainly forms oligomeric complexes through intermolecular disulfide linkages. Following pathogen infection, SA accumulation changes the cellular redox status and promotes the conversion of NPR1 oligomers into monomeric forms (Mou et al., 2003). The resulting NPR1 monomers enter the nucleus and associate with TGA transcription factors to induce the expression of *PATHOGENESIS-RELATED* (*PR*) genes, which contributes to enhanced pathogen resistance (Backer et al., 2019).

ROS serve important roles in plant immune signaling. Following pathogen invasion, plants rapidly accumulate ROS molecules, such as superoxide (O_2_^-^) and hydrogen peroxide (H_2_O_2_) (Bindschedler et al., 2006; Jia et al., 2010; An et al., 2021). These molecules contribute directly to defense through multiple mechanisms, such as strengthening the cell wall barrier and disrupting pathogen membrane integrity (Torres et al., 2006). Furthermore, H_2_O_2_ functions as an important signaling mediator that activates defense-related gene expression and facilitates hypersensitive cell death at infection sites, collectively enhancing pathogen restriction (Apostol et al., 1989; Hammond-Kosack and Jones, 1996; Torres et al., 2006). Moreover, ROS signaling operates in close coordination with SA-dependent pathways and other immune regulators (Torres et al., 2006), thereby forming an integrated network that fine-tunes immune responses.

In this study, we investigate the contribution of resveratrol monomer to *Arabidopsis* resistance against *Pst* DC3000, which remains insufficiently understood. We demonstrate that resveratrol enhances pathogen resistance independently of direct antibacterial activity. We further show that resveratrol promotes SA accumulation, induces NPR1 nuclear translocation, activates *PR* gene expression, and stimulates ROS production. These findings establish a previously uncharacterized role for resveratrol monomer as a regulator of host defense signaling and redefine its function beyond conventional antimicrobial activity.

## Materials and methods

### Plant materials and growth conditions

The wild-type accession used in this study was *Arabidopsis thaliana* Columbia-0 (Col-0) were used in this study. All mutants and transgenic plants were derived from the Col-0 genetic background. The mutants plants *sid2* (Wildermuth et al., 2001), *npr1-1* (Cao et al., 1997), *npr3-2* (Fu et al., 2012), and *npr4-2* (Fu et al., 2012), together with the transgenic plants *NahG* (Lawton et al., 1995) and *35S::NPR1-GFP/npr1-2* (*35S::NPR1-GFP OX*; Spoel et al., 2009), were used for the experiments.

Seeds were sterilized by soaked in 70% ethanol for 1 min, and subsequently treated with 10-fold dilution of commercial bleach solution (0.4% NaOCl) for 10 min. After four rinses with sterile distilled water, seeds were sown on Murashige Skoog (MS) medium (Murashige and Skoog, 1962) supplemented with vitamins, 2.0% sucrose, and 0.8% agar. Following a 3-d stratification period, seedlings were cultivated under a 16-h light/8-h dark photoperiod with a light intensity of approximately 120 μmol m^-2^ s^-1^ at 22°C. For bacterial growth assays, two-week-old plants were transferred to soil and grown for additional 3 weeks under an 8-h light/16-h dark cycle at 24°C with the same light intensity.

### Analysis of antimicrobial activity

To measure antimicrobial activity on solid medium, we followed a previously described method (An et al., 2021). *Pst* DC3000 was cultured on *Pseudomonas* agar F medium (MB cell, Korea) supplemented with rifampicin (30 mg/L) at 28°C for 48 h. Bacterial cells were suspended in 10 mM MgCl_2_ and adjusted to concentrations of 10^5^, 10^6^, 10^7^, and 10^8^ colony-forming units (cfu)/mL. Aliquots of each suspension were spotted onto King’s B (KB) agar plates containing 0, 50, 100, and 200 μM resveratrol (R5010, Sigma-Aldrich, USA). After incubation at 28°C for 36 h, the plates were photographed.

To evaluate antibacterial activity of resveratrol in liquid medium, we analyzed the growth curve of *Pst* DC3000 as previously described (Kang et al., 2020b; Bredow et al., 2025). Overnight liquid-cultures of *Pst* DC3000 was diluted to 1 x 10^7^ cfu/mL, and 1 mL of the bacterial suspension was inoculated into 100 mL of KB broth supplemented with antibiotics (kanamycin and rifampicin) and various concentrations of resveratrol (0, 50, 100, 200, and 500 μM). Cultures were maintained at 28°C for 24 h. Samples were collected every 2 h, and bacterial growth was monitored by measuring optical density at 600 nm (OD_600_) using a spectrophotometer. OD_600_ values were used to estimate bacterial population density.

### Bacterial growth assays

Bacterial growth assays with the *Pst* DC3000 were performed as previously described (Gassmann, 2005). Five-week-old plants grown in soil were pre-infiltrated with 100 µM resveratrol for 2 days. The leaves were then inoculated with a bacterial suspension containing 5 × 10^5^ cfu/mL in 10 mM MgCl_2_ using a 1 mL needleless syringe. Leaf discs (total area 1 cm^2^) were collected from the infected tissues at 3 or 5 days post-inoculation (dpi). The samples were homogenized in 10 mM MgCl_2_, serially diluted, and plated on King’s B agar medium supplemented with kanamycin and rifampicin antibiotics. Bacterial colonies were counted after incubation at 28°C for 2 d, and bacterial growth was expressed as cfu/cm^2^ of leaf tissue.

### RNA isolation and RT-qPCR analysis

Total RNA was isolated from 14-day-old plants grown on MS medium using a TRIzol^®^ reagent (Invitrogen, USA), according to the provided protocol. First-strand cDNA synthesis was performed using 5 μg of total RNA and RevertAid Reverse Transcriptase (Invitrogen, USA). Quantitative PCR (qPCR) was conducted with iQ SYBR^®^ Green Supermix (Bio-Rad, USA) on a CFX384 Real-Time PCR System (Bio-Rad). Primer sequences used in this study are listed in **Supplementary Table S1**. The amplification program consisted of an initial pre-denaturation step at 94°C for 5 min, followed by 45 cycles of 94°C for 45 s (denaturation), 55°C for 30 s (annealing), and 72°C for 40 s (extension). Melting-curve analysis was subsequently performed over 90 cycles with incremental temperature increases of 0.5 °C per cycle beginning at 55°C. A final extension step was carried out 72 °C for 10 min.

### Confocal microscopy

Two-week-old *35S::NPR1-GFP OX* plants were applied with mock, 100 μM SA, or 100 μM resveratrol for 24 h. Leaf samples were mounted in distilled water and observed using an Olympus FV1000 confocal laser-scanning microscope. GFP florescence was exited at 488 nm, and emission signals were collected between 500 and 539 nm. Images were acquired a detector gain value of 556.

### Cytoplasmic and nuclear fractionation

Two-week-old *35S::NPR1-GFP OX* plants were treated with 100 μM resveratrol for 24 h and harvested for subcellular fractionations. Cytoplasmic and nuclear fractions were isolated using the CelLytic™ PN Isolation/Extraction Kit (Sigma-Aldrich) according to the manufacturer’s instructions. NPR1, phosphoenolpyruvate carboxylase (PEPC), and histone H3 (H3) were detected by immunoblot analysis using anti-NPR1 (1:5,000 dilution; Abiocode, USA), anti-PEPC (1:10,000 dilution; Abcam, UK), and anti-H3 (1:10,000 dilution; Abcam) antibodies, respectively. PEPC and H3 were used as cytoplasmic and nuclear marker proteins, respectively.

### Measurement of SA levels

Endogenous SA was quantified using a modified QuEChERS-based extraction procedure (Anastassiades et al., 2003). Two-week-old plants were treated with resveratrol for 24 h, frozen in liquid nitrogen, and finely powdered. Approximately 50 mg of tussore powder was transferred to a 1.5 mL microcentrifuge tube and extracted with 1 mL of 80% acetonitrile containing 1% formic acid. After homogenized for 2 min. samples were centrifuged at 4 °C for 10 min. The resulting supernatant was subjected to salt-assisted partitioning in a 2 mL tube containing 50 mg MgSO_4_ and 25 mg NaOAc. Following vortexing for 1 min and centrifugation at 13,000 rpm for 5 min, the organic phase was collected and further cleaned using dispersive solid-phase extraction with 25 mg C18 and 150 mg MgSO_4_. The mixture was vortexed for 1 min and centrifuged at 13,000 rpm for 5 min, after with the clarified extract was passed through a 0.22 μm PTFE syringe membrane filter (SUPERFIL, Korea).

Chromatographic analysis was conducted using a Nexera X2 UHPLC system (Shimadzu Corporation, Japan). Aliquots (2 μL) were injected onto a Waters ACQUITY BEH C18 UHPLC column (2.1 x 100 mm, 1.7 µm particle size) maintained at 40 °C. Separation employed a binary solvent system consisting of 0.1% formic acid in water (solvent A) and 0.1% formic acid in acetonitrile (solvent B) at a flow rate of 0.3 mL/min. The gradient profile was program as follow: 10% B at 0 min, 90% B at 7 min, and 90% B at 10 min.

Detection of SA was carried out using a QTRAP 4500 triple quadrupole/linear ion trap mass spectrometer (SCIEX, Framingham, MA, USA) operated in negative electrospray ionization mode at the High-Tech Materials Analysis Core Facility (Jinju, Korea; NFEC-2019-03-255243). Data acquisition employed multiple reaction monitoring (MRM). Instrument settings included on an ion spray voltage of -4500 V, curtain gas of 30 psi, ion source gas1 of 50 psi, and a source temperature of 500 °C. Collision-induced dissociation was conducted at -22 eV. SA was monitored using the transition *m/z* 137 → 97 with a declustering potential of -5 V. Quantification was based on an external calibration curve generated from authentic SA standards. Peak integration and data processing were performed using SCIEX OS software (version 1.4.0.18607), and SA concentrations were expressed as ng/g dry weight (ng/g DW).

### Histochemical staining and quantification of hydrogen peroxide (H_2_O_2_)

H_2_O_2_ accumulation was analyzed by both histochemical staining and quantitative fluorescence assays. For histochemical detection, two-week-old plants were exposed to mock, 100 µM SA, or 100 µM resveratrol for 24 h. H_2_O_2_ accumulation was visualized using 3,3’-diaminobenzidine (DAB; Sigma-Aldrich a) as previously described (Thordal-Christensen et al., 1997). Leaves were immersed in 1 mg/mL DAB solution for 8 h and subsequently cleared in bleaching solution containing ethanol, acetic acid, and glycerol (3:1:1, v/v/v) at 90°C for 10 min. Stained tissues were photographed under light microscope.

For quantitative determination of H_2_O_2_, the Amplex™ Red Hydrogen Peroxide/Peroxidase Assay Kit (Thermo Fisher Scientific, USA) was used with minor modifications as previously described (Zhou et al., 1997; Brumbarova et al., 2016). Approximately 0.1 g of frozen plant tissue was ground to a fine powder in liquid nitrogen and homogenized in 500 μL of 20 mM potassium phosphate buffer (K_2_HPO_4_, pH 6.5). After centrifugation at 12,000 rpm for 15 min at 4 °C, the supernatant was collected for analysis. Fifty microliters of the extract were mixed with an equal volume of freshly prepared reaction mixture containing 1× reaction buffer, 10 U/mL horseradish peroxidase (HRP), and 10 mM Amplex^®^ Red reagent. After incubation for 30 min at room temperature in the dark, fluorescence was measured using a GloMax^®^ Discover Microplate Reader (Promega, USA) with excitation and emission wavelengths of 530 and 590 nm, respectively. H_2_O_2_ concentrations were calculated from a standard curve generated using known concentrations of H_2_O_2_.

### Protein extraction and immunoblot analysis

Protein samples were prepared from 2-week-old plants treated with mock or resveratrol. Plant tissues were homogenized in extraction buffer containing 50 mM Tris-HCl (pH 7.5), 5 mM EDTA, 5 mM EGTA, 1 mM Na_3_VO_4_, 25 mM NaF, 50 mM *β*-glycerophosphate, 2 mM DTT, 2 mM PMSF, 5% glycerol, 1% Triton X-100, and protease inhibitor cocktail. Equal amounts of protein were resolved by SDS-PAGE and transferred for immunoblot analysis. Phosphorylated MPK3 and MPK6 were detected using anti-phospho-p44/42 MPK antibodies (1:5000 dilution; Cell Signaling Technology, USA). PR1 and NPR1 proteins were detected using anti-PR1 (1:10,000 dilution; Agrisera, Sweden) and anti-NPR1 (1:5,000 dilution; Abiocode) antibodies, respectively.

### Statistical analysis

Statistical analysis was carried out using GraphPad Prism 9 software (GraphPad Software, USA). Each experiment included three biological replicates and was repeated independently three times. Statistical significance was evaluated using a two-tailed Student’s *t*-test or two-way ANOVA followed by Tukey’s *post hoc* test. Differences were considered significant at P < 0.05. Data are presented as means ± standard deviation (SD).

## Results

### Resveratrol enhances resistance to *Pst* DC3000 in *Arabidopsis*

Resveratrol, including its monomeric form, has been characterized primarily as a phytoalexin with direct antimicrobial activity (Chan, 2002; Vestergaard and Ingmer, 2019; Bejenaru et al., 2024; Li et al., 2024). However, it remains unclear whether resveratrol monomer (hereafter referred to as resveratrol) enhances host resistance through modulation of endogenous immune signaling. Recent studies have reported that flavonoids, another class of phytoalexins, participate in plant immune regulation in addition to their antimicrobial functions (Jia et al., 2010; An et al., 2021, 2022, 2023). Based on these findings, we investigated whether resveratrol similarly contribute to pathogen resistance through activation of host immune responses. To evaluate this possibility, we examined the effect of resveratrol on disease symptoms in *Arabidopsis* Col-0 plants infected with *Pst* DC3000. At 3 days post-inoculation (dpi), mock-treated Col-0 plants exhibited pronounced chlorosis, whereas plants treated with 100 µM resveratrol showed reduced disease symptoms (**Figure 1A**). This difference became more apparent at 5 dpi. Consistent with these observations, bacterial growth was significantly reduced in resveratrol-treated plants related to the mock-treated controls (**Figure 1B**). These results indicate that resveratrol increases *Arabidopsis* resistance against *Pst* DC3000.

**Figure 1:**
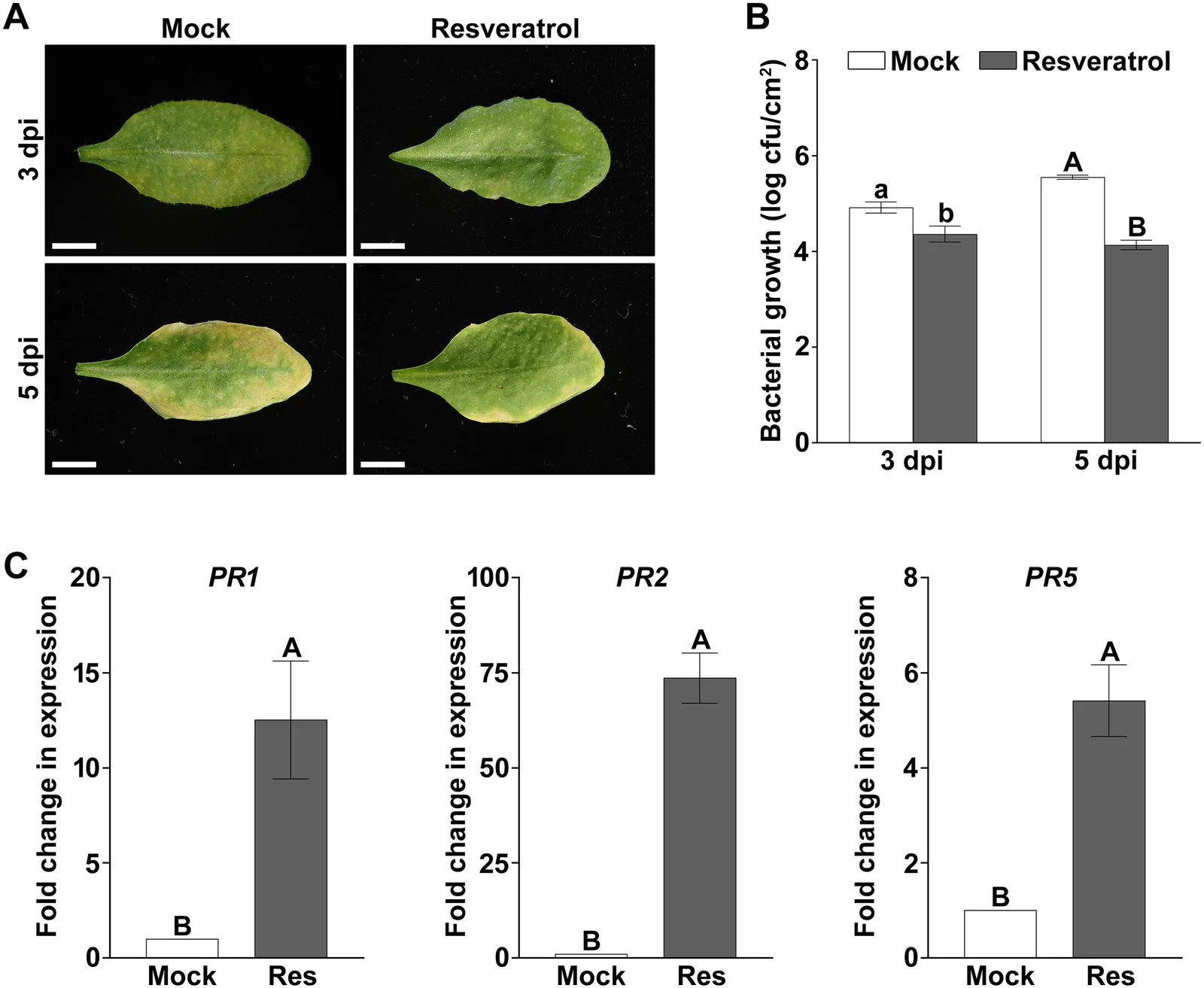
Resveratrol enhances resistance against *Pst* DC3000 in *Arabidopsis*. **(A)** Effect of resveratrol on disease symptoms in Col-0 plants inoculated with *Pst* DC3000. Plants were pretreated with mock or 100 μM resveratrol for 2 d and then infiltrated with *Pst* DC3000 (5 × 10^5^ cfu/mL). Photographs were taken at 3 or 5 dpi (days post inoculation). Scale bar = 0.5 cm. **(B)** Suppression of bacterial growth by resveratrol. Bacterial growth was quantified by counting colony-forming units (cfu) at 3 or 5 dpi, as shown in **(A)**. Each biological replicate consisted of three leaf discs collected from different leaves of the same plant. Bars represent the mean ± S.D. (*n* = 4). Different letters indicate statistically significant differences, as determined by two-way ANOVA with Tukey’s *post hoc* test (*P* < 0.001). **(C)** Transcriptional upregulation of *PR* genes by resveratrol treatment. Total RNA was extracted from Col-0 plants treated with mock or 100 µM resveratrol (Res) for 24 h. Transcript levels of *PR1*, *PR2*, and *PR5* were measured by RT-qPCR using specific primers. *Tubulin* was used as an internal control. Bars represent the mean ± S.D. (*n* = 3). Different letters indicate statistically significant differences, as determined by Student’s *t*-test (*P* < 0.01).

To determine whether this enhanced resistance resulted from direct antimicrobial activity of resveratrol, we performed bacterial growth assays on solid medium supplemented with resveratrol at various concentrations. The assays showed that no significant inhibition of bacterial proliferation was observed at concentrations up to 200 µM resveratrol (**Supplementary Figure 1A**). Similar results were obtained in liquid culture assays. Although bacterial growth was obviously reduced at 500 µM resveratrol, concentration up to 200 µM had no detectable effect on bacterial proliferation (**Supplementary Figure 1B**). These results indicate that resveratrol does not exert direct antibacterial activity against *Pst* DC3000 under our experimental conditions (100 µM resveratrol). Therefore, the reduced disease symptoms and bacterial growth observed in resveratrol-treated plants are attributable to enhanced host resistance rather than to a direct antimicrobial effect.

To elucidate the underlying mechanism of resveratrol-induced resistance, we analyzed the transcript abundance of the SA-associated defense marker genes *PR1*, *PR2*, and *PR5*, by RT-qPCR after resveratrol treatment. As a result, resveratrol strongly elevated the expression of these genes by approximately 12.5-, 73.6-, and 5.4-fold, respectively, compared to mock-treated plants (**Figure 1C**). These findings demonstrate that resveratrol stimulates SA-related defense signaling and promotes the transcriptional activation of key immune-responses genes in *Arabidopsis*.

### NPR1 mediates resveratrol-induced resistance to bacterial infection

NPR1 functions as a central regulator of SA-mediated immunity and activates the transcription of *PR* genes. Therefore, we first assessed whether NPR1 is required for resveratrol-induced resistance. To address this question, we analyzed disease symptoms and bacterial proliferation in the *npr1–1* mutants and *35S::NPR1-GFP OX* plants. Compare to Col-0 plants, *npr1–1* mutants failed to exhibit reductions in chlorotic symptoms or bacterial growth following resveratrol treatment (**Figures 2A, B**). Furthermore, resveratrol-induced expression of *PR* genes was completely abolished in the mutant background (**Figure 2C**). In contrast, *35S::NPR1-GFP OX* plants showed enhanced resistance to bacterial infection regardless of resveratrol treatment, and no further increase in resistance was observed after resveratrol application (**Figure 2**). Importantly, resveratrol-treated Col-0 plants exhibited a level of resistance comparable to that of *35S::NPR1-GFP OX* plants. These results indicate that NPR1 is essential for resveratrol-mediated resistance.

**Figure 2:**
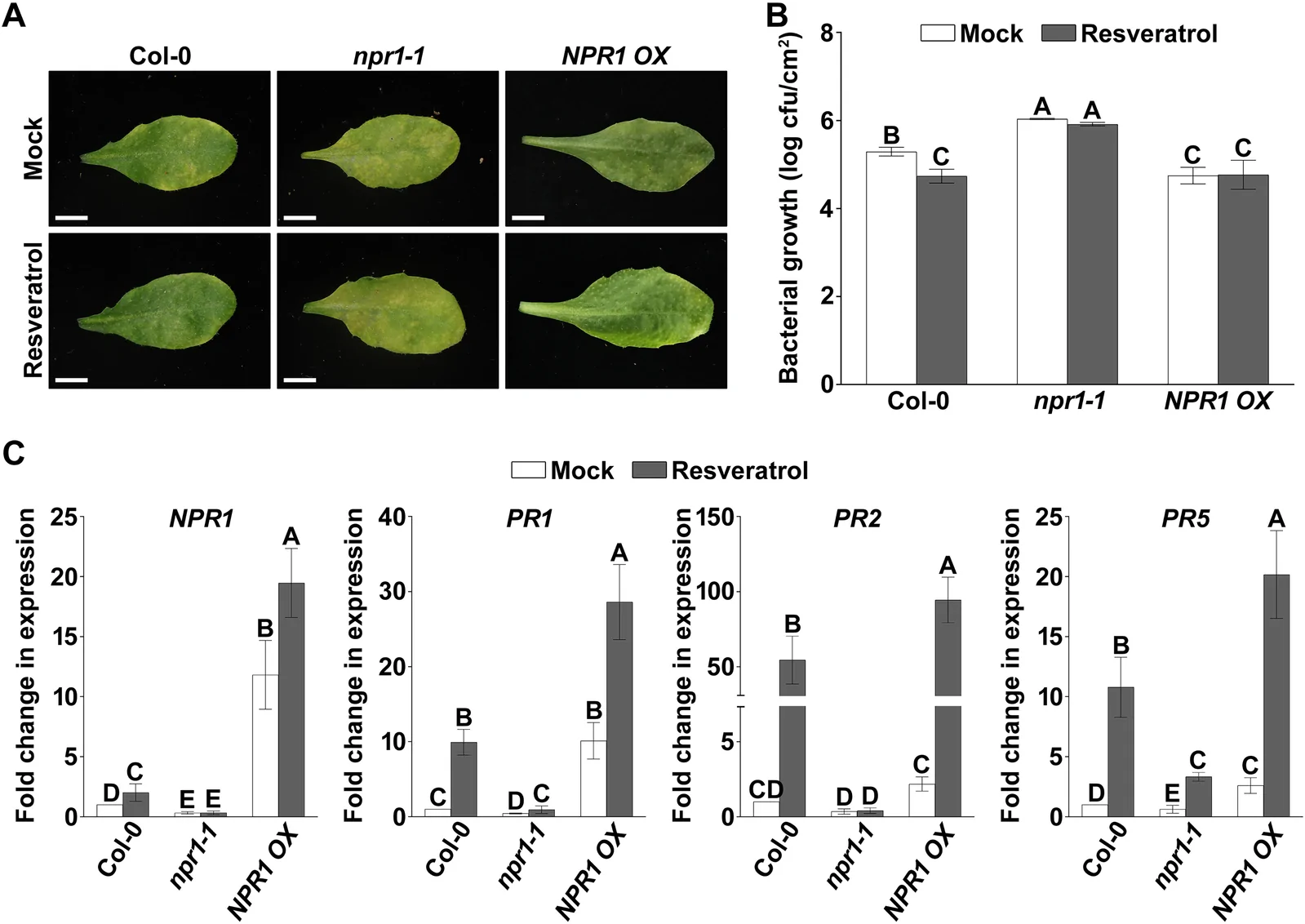
NPR1 is required for resveratrol-induced resistance to bacterial pathogens. **(A)** Comparison of disease symptoms in Col-0, *npr1-1*, and *35S::NPR1-GFP OX* (*NPR1 OX*) plants treated with resveratrol. Plants were pretreated with mock or 100 μM resveratrol for 2 d and then inoculated with *Pst* DC3000 (5 × 10^5^ cfu/mL). Photographs were taken at 3 dpi. Scale bar = 0.5 cm. **(B)** Reduced suppression of bacterial growth by resveratrol is abolished in the *npr1–1* mutant. Bacterial growth was measured by counting cfu at 3 dpi, as shown in **(A)**. Each biological replicate consisted of three leaf discs collected from different leaves of the same plant. Bars represent the mean ± S.D. (*n* = 4). Different letters indicate statistically significant differences, as determined by two-way ANOVA with Tukey’s *post hoc* test (*P* < 0.001). **(C)** Resveratrol-induced expression of *PR* genes is required for NPR1. Total RNA was extracted from Col-0, *npr1-1*, and *35S::NPR1-GFP OX* plants treated with mock or 100 µM resveratrol (Res) for 24 h. Transcript levels of *PR1*, *PR2*, and *PR5* were measured by RT-qPCR using specific primers. Bars represent the mean ± S.D. (*n* = 3). Different letters indicate statistically significant differences, as determined by one-way ANOVA with Tukey’s *post hoc* test (*P* < 0.001).

Next, to investigate how resveratrol regulates NPR1, we analyzed NPR1 expression. As a result, although *PR1* expression was increased in a time-dependent manner after resveratrol treatment, transcript levels of *NPR1* did not change (**Supplementary Figures 2A, B**). Similar pattern was observed at the protein levels. Resveratrol increased PR1 protein accumulation but did not alter NPR1 protein abundance (**Supplementary Figure 3**). These results suggest that resveratrol may regulate NPR1 through a post-translation mechanism rather than through transcriptional regulation, which is consistent with the established mode of NPR1 regulation.

Upon pathogen challenge, changes in cellular redox status convert NPR1 oligomers into monomers, which subsequently accumulate in the nucleus in response and activate the transcription of *PR* genes (Maier et al., 2011; Wu et al., 2012). Given this regulatory mechanism, we investigated whether resveratrol promotes the nuclear translocation of NPR1, thereby inducing *PR* gene expression. To address this question, the subcellular localization of GFP-tagged NPR1 was examined in *35S::NPR1-GFP OX* plants after resveratrol treatment. Under mock conditions, GFP signals were predominantly observed in the cytoplasm, with only weak nuclear signals in stomatal guard cells (**Figure 3A**). In contrast, strong nuclear GFP fluorescence was observed after resveratrol treatment, similar to the pattern observed after SA treatment. These results indicate that resveratrol promotes the accumulation of NPR1 in the nucleus.

**Figure 3:**
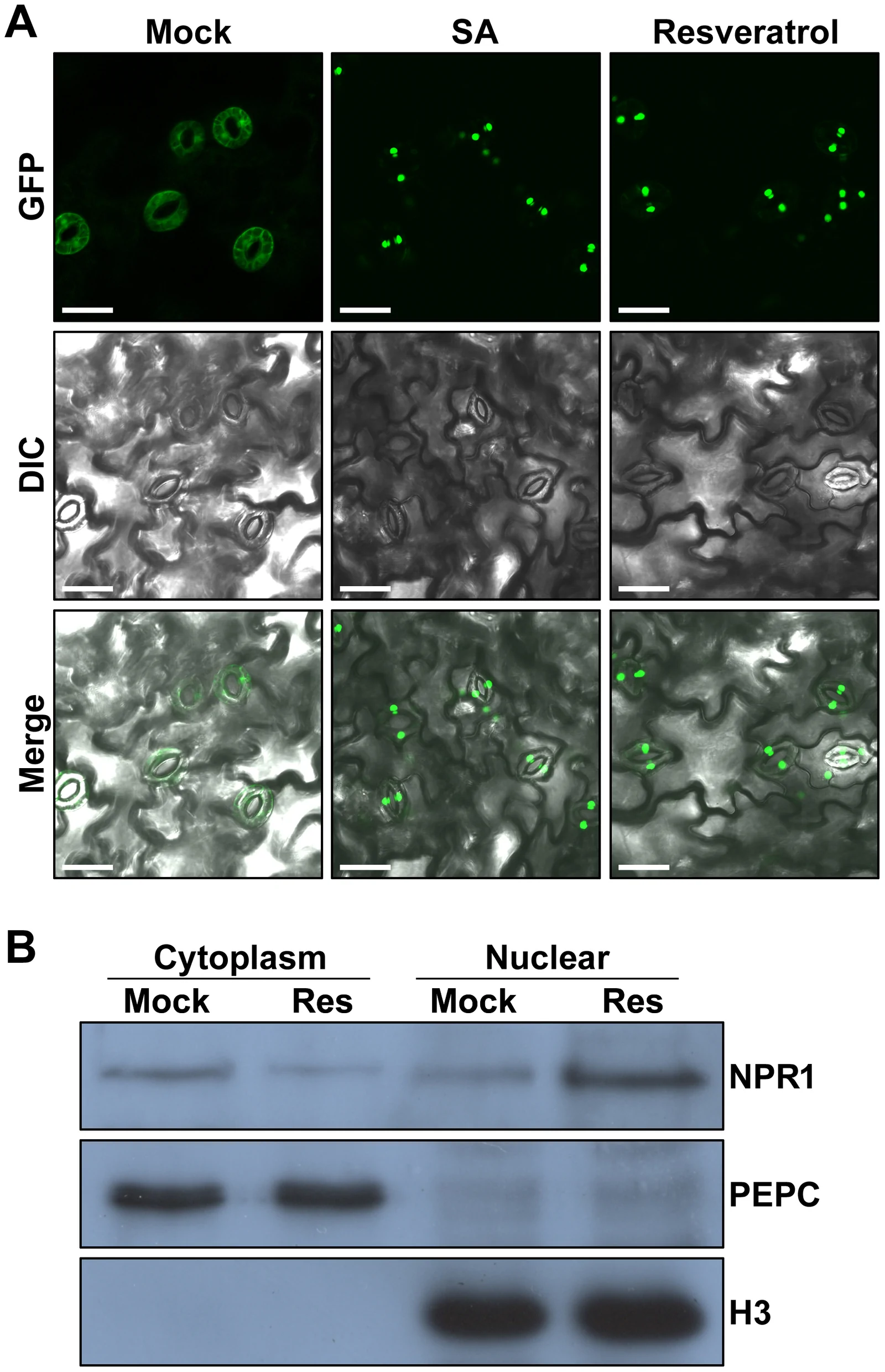
Resveratrol promotes the nuclear translocation of NPR1. **(A)** Resveratrol promotes nuclear localization of NPR1 in guard cells. *35S::NPR1-GFP OX* plants were treated with mock, 100 μM SA, or 100 μM resveratrol for 24 h. NPR1-GFP fluorescence in guard cells was observed using a laser scanning confocal microscope. Representative images for each treatment are shown. Scale bars = 30 μm. Similar results were obtained in three independent experiments. **(B)** Resveratrol enhances NPR1 accumulation in the nucleus. Total proteins from *35S::NPR1-GFP OX* plants treated with 100 μM resveratrol were fractionated into cytoplasmic and nuclear fractions. The protein abundance of NPR1 was determined by immunoblot analysis using anti-NPR1 antibodies. PEPC and H3 were used as markers for the cytoplasmic and nuclear fractions, respectively.

To further validate this observation, we performed subcellular fractionation analysis using *35S::NPR1-GFP OX* plants following resveratrol treatment. Consistent with the microscopy results, resveratrol markedly increased NPR1 protein levels in the nuclear fraction compared to the mock treatment (**Figure 3B**). These results demonstrate that resveratrol promotes the nuclear translocation and accumulation of NPR1, which likely contributes to the activation of SA-dependent defense responses. Collectively, these findings demonstrate that NPR1-dependent signaling is indispensable for resveratrol-enhanced resistance to bacterial pathogens.

NPR3 and NPR4 have also been identified as SA receptors and function antagonistically to NPR1 in SA-mediated defense signaling (Fu et al., 2012; Ding et al., 2018). To further investigate their role within the NPR signaling network during resveratrol-induced resistance, we examined the effects of resveratrol on *NPR3* and *NPR4* expression and function. RT-qPCR analysis showed that the transcript levels of *NPR3* and *NPR4* did not change significantly after resveratrol treatment, although a slight induces was observed after 12 h (**Supplementary Figures 2C, D**). In addition, bacterial growth assays showed that resveratrol significantly reduced pathogen proliferation in *Col-0*, *npr3-2*, and *npr4–2* plants compared with the corresponding each mock-treated plant. Importantly, no significant differences in bacterial growth were observed among three genotypes following resveratrol treatment (**Supplementary Figure 4**). These results indicated that neither NPR3 nor NPR4 appears to be individually involved in the resveratrol-mediated pathogen resistance. Therefore, the experiments in *npr3 npr4* double mutant were not further examined. Collectively, these results suggest that NPR3 and NPR4 play limited roles in resveratrol-induced resistance under our experimental conditions, whereas NPR1 functions as a key positive regulator of this response.

### Resveratrol enhances pathogen resistance through an SA-dependent pathway

SA is a key hormone that mediates defense responses against biotrophic and hemibiotrophic pathogens. Upon bacterial infection, SA is accumulated at the infection site and promotes resistance (An and Mou, 2011; Zhu et al., 2024). To investigate whether SA participates in the defense response triggered by resveratrol, we quantified endogenous SA levels in Col-0 plants after resveratrol treatment. Notably, resveratrol treatment increased endogenous SA levels by approximately 3.5-fold compared with mock treatment (**Figure 4A**), indicating that resveratrol promotes SA accumulation in plants.

**Figure 4:**
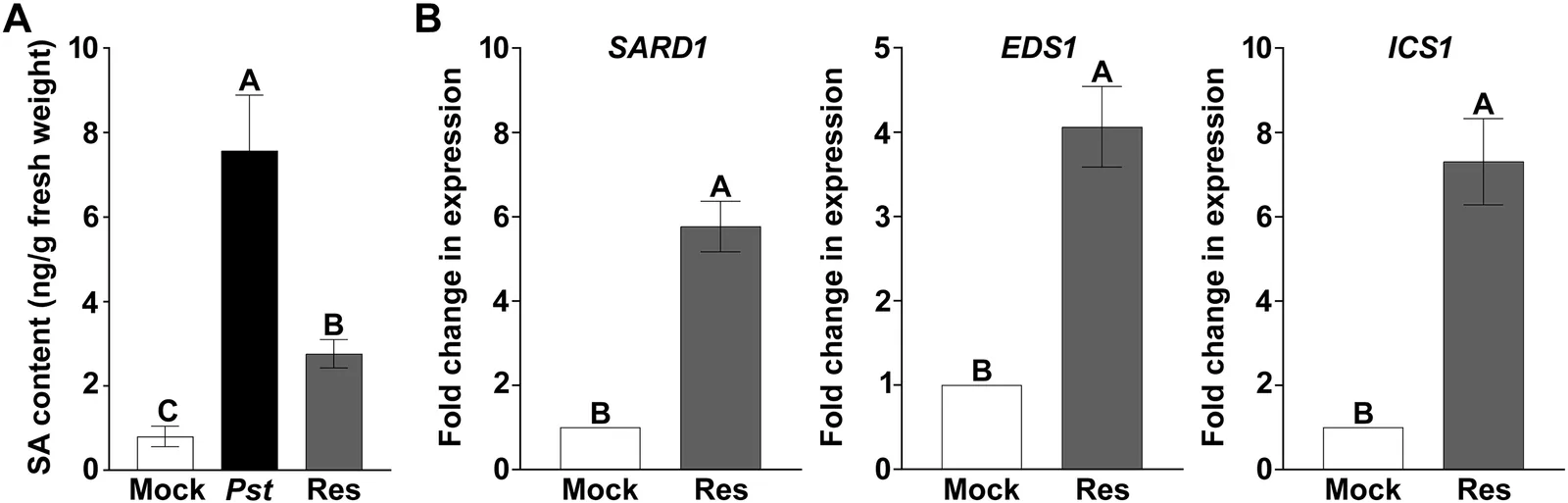
Resveratrol increases SA accumulation through transcriptional activation of SA biosynthesis genes. **(A)** Resveratrol elevates endogenous SA levels in plants. SA content was quantified by LC-MS/MS after treatment with mock, *Pst* DC3000 (*Pst*), or 100 µM resveratrol (Res) for 24 h. Bars represent the mean ± S.D. (*n* = 3). Different letters indicate statistically significant differences, as determined by Student’s *t*-test (*P* < 0.01). **(B)** Transcriptional upregulation of SA biosynthesis genes by resveratrol treatment. Total RNA was extracted from Col-0 plants treated with mock or 100 µM resveratrol (Res) for 24 h. Transcript levels of *SARD1*, *EDS1*, and *ICS1* were quantified by RT-qPCR using specific primers. *Tubulin* was used as an internal control. Bars represent the mean ± S.D. (*n* = 3). Different letters indicate statistically significant differences, as determined by Student’s *t*-test (*P* < 0.001).

To explore the basis of this increase, we examined the expression of genes associated with SA production, including *SARD1*, *EDS1*, and *ICS1*. As expected, resveratrol treatment increased the transcript levels of these genes by approximately 5.7-, 4.0-, and 7.3-fold, respectively (**Figure 4B**). These findings suggest that resveratrol enhances SA accumulation through activation of SA biosynthetic pathway.

We then evaluated whether resveratrol-induced resistance depends on SA signaling. Disease symptoms and bacterial proliferation were analyzed in the SA-deficient *sid2* mutant and *NahG* transgenic plants. Unlike Col-0 plants, resveratrol-induced resistance was markedly reduced in both *sid2* and *NahG* plants (**Figures 5A, B**). Furthermore, resveratrol failed to induce *PR1* expression in both plants (**Figure 5C**). Together, these findings demonstrate that the protective effect of resveratrol against bacterial infection relies largely on an intact SA signaling pathway.

**Figure 5:**
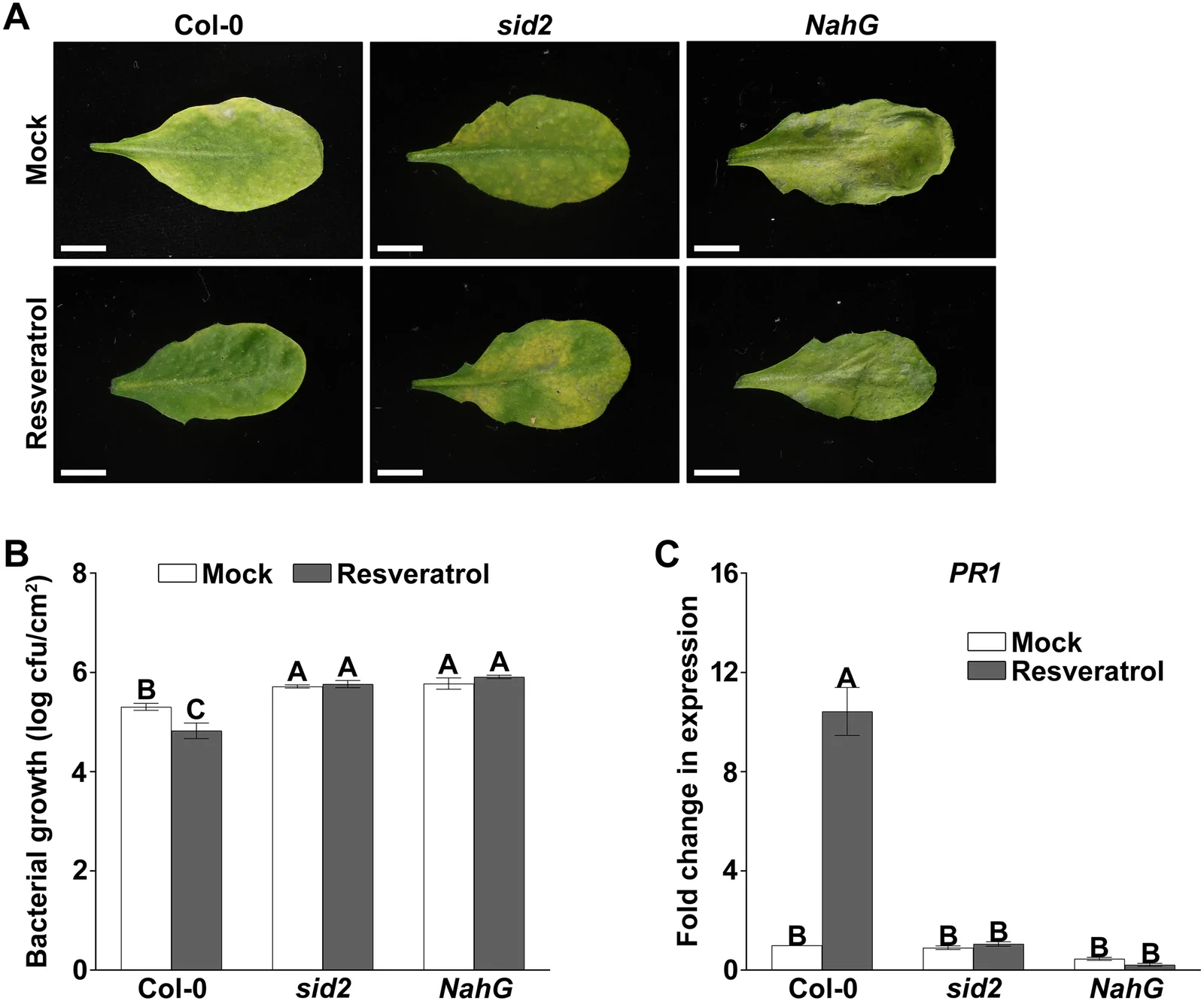
Resveratrol-induced pathogen resistance depends on SA signaling. **(A)** Comparison of disease symptoms in resveratrol-treated Col-0 and SA-deficient mutants following *Pst* DC3000 infection. Plants were pretreated with mock or 100 µM resveratrol for 2 d and then infiltrated with *Pst* DC3000 (5 × 10^5^ cfu/mL). Symptoms were recorded at 3 dpi. **(B)** Impaired suppression of bacterial growth by resveratrol in Col-0, *sid2*, and *NahG* plants. Bacterial growth was measured by counting cfu at 3 dpi, as shown in **(A)**. Bars represent the mean ± S.D. (*n* = 4). Different letters indicate statistically significant differences among genotypes, as determined by two-way ANOVA with Tukey’s *post hoc* test (*P* < 0.001). **(C)** Reduced resveratrol-induced *PR1* expression in *sid2* and *NahG* plants. Total RNA was extracted from Col-0, *sid2*, and *NahG* plants treated with mock or 100 µM resveratrol for 24 h. Transcript levels of *PR1* gene were quantified by RT-qPCR using specific primers. *Tubulin* was used as an internal control. Bars represent the mean ± S.D. (*n* = 3). Different letters indicate statistically significant differences among genotypes, as determined by two-way ANOVA with Tukey’s *post hoc* test (*P* < 0.001).

### Resveratrol induces ROS accumulation

The ROS generation represents one of the earliest cellular responses to pathogen invasion and plays an important role in immune signaling (Apel and Hirt, 2004; Tsuda and Katagiri, 2010). Among these molecules, H_2_O_2_ contributes not only to antimicrobial defense but also to the activation of downstream defense-related gene expression (Apel and Hirt, 2004). To investigate whether resveratrol triggers ROS production, we monitored H_2_O_2_ accumulation by histochemical 3,3′-diaminobenzidine (DAB) staining. Leaves treated with resveratrol exhibited strong DAB staining, indicating substantial H_2_O_2_ accumulation. The intensity of staining was comparable to that observed in SA-treated plants (**Figure 6A**). Consistent with DAB staining results, H_2_O_2_ quantitative analysis showed that H_2_O_2_ levels were significantly higher in resveratrol-treated plants than in mock-treated plants (**Figure 6B**). These results demonstrate that resveratrol enhances ROS production, which may contribute to the activation of downstream defense signaling pathway in *Arabidopsis*.

**Figure 6:**
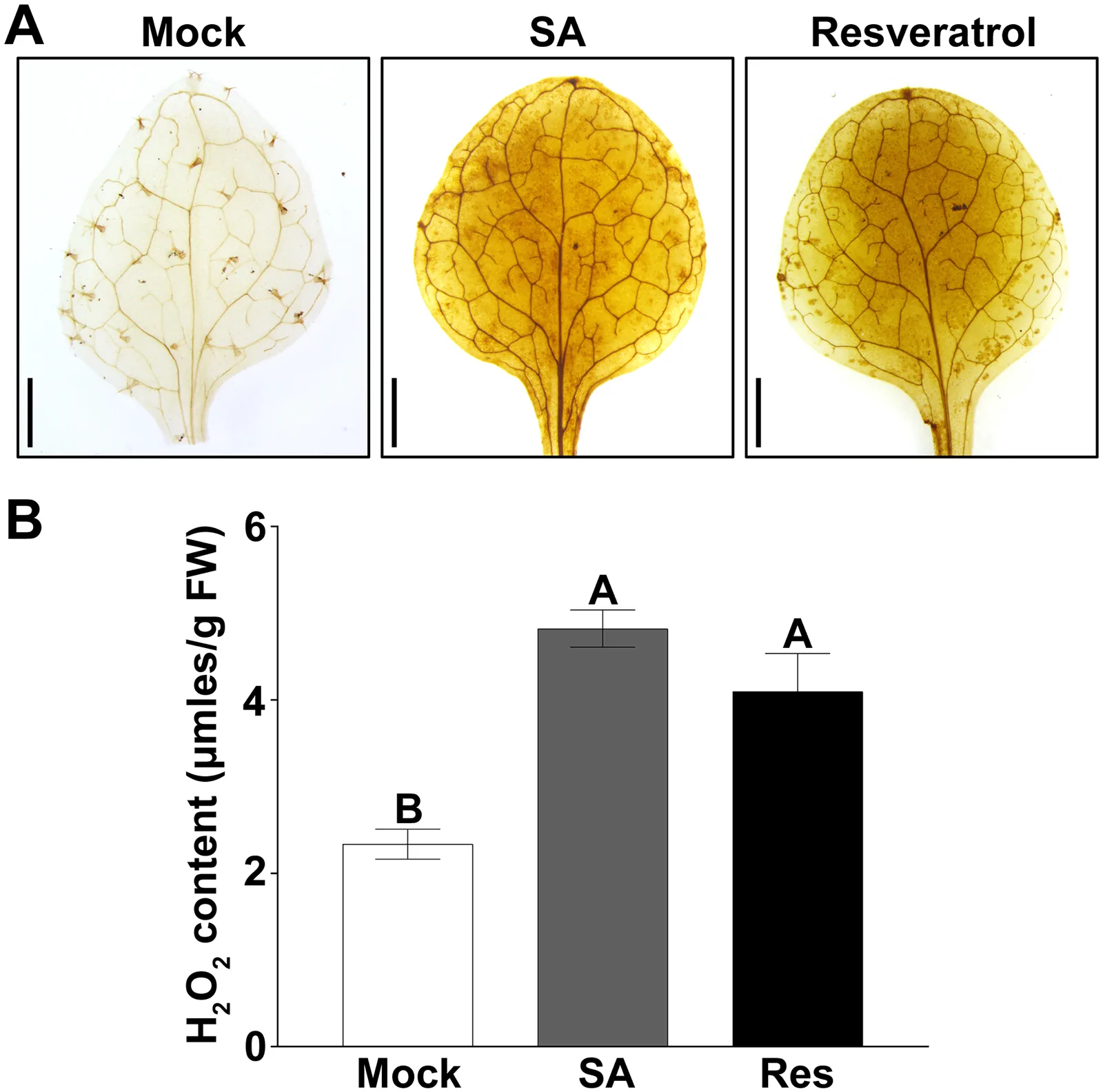
Resveratrol induces accumulation of reactive oxygen species (ROS). **(A)** Histochemical DAB staining for H_2_O_2_ production. Col-0 plants were treated with mock, 100 µM SA or 100 µM resveratrol for 24 h. H_2_O_2_ accumulation in leaves was visualized by DAB staining. Representative images for each treatment are shown. Scale bar = 1 mm. Similar results were obtained in three independent experiments. **(B)** Quantitative measurement of H_2_O_2_ accumulation using the Ameplex Red. Col-0 plants were treated with mock, 100 µM SA, 100 µM resveratrol for 24 h before H_2_O_2_ quantification. Bars represent the mean ± S.D. (*n* = 3). Different letters indicate statistically significant differences among treatments, as determined by one-way ANOVA with Tukey’s *post hoc* test (P < 0.001).

## Discussion

### Resveratrol functions as an immune activator in *Arabidopsis*

Resveratrol has been widely recognized as a phytoalexin with antimicrobial activity. However, accumulating evidence indicates that plant secondary metabolites, including phytoalexins also function as inducers of immune signaling (Zhang et al., 2009; Jia et al., 2010; Jeandet et al., 2013; Vestergaard and Ingmer, 2019; An et al., 2023). In this study, we demonstrate that exogenous resveratrol application significantly reduced disease symptoms and suppressed *Ps*t DC3000 growth in *Arabidopsis* (**Figure 1**). Since resveratrol did not inhibit bacterial growth *in vitro* at the tested concentrations, the enhanced resistance observed *in planta* is unlikely to result from a direct antibacterial effect. These results indicate that resveratrol enhances resistance through activation of endogenous defense mechanisms. Similar observations have been reported in other plant species. In wheat, exogenous application of resveratrol enhanced resistance to powdery mildew and activated defense-related metabolism (Pociecha et al., 2014). Likewise, Meng et al. (2025) reported that transient expression of a peanut resveratrol synthase homolog enhanced resistance of *Nicotiana benthamiana* to *Botrytis cinerea*, indicating that resveratrol biosynthesis contributes to plant immune responses. Together with our findings, these results strongly support the role of resveratrol as a regulator of plant immunity.

Early immune signaling in plants commonly involves rapid activation of mitogen-activated protein kinase (MPK) cascades (Meng and Zhang, 2013). Notably, we observed that MPK3 and MPK6 were activated within 5 min after resveratrol treatment (**Supplementary Figure 5A**), indicating that resveratrol triggers early immune signaling events. This rapid MPK activation suggests that resveratrol functions upstream of, or in parallel with, canonical immune signaling pathways rather than acting solely as an antimicrobial compound. Collectively, these findings establish resveratrol as a signaling-active metabolite that modulates plant immune responses in addition to its antimicrobial function.

### Resveratrol induces pathogen resistance through the SA-mediated NPR1 pathway

NPR1 is a central regulator of resistance to bacterial pathogens and requires nuclear translocation for activation (An and Mou, 2011; Klessig et al., 2018). In this study, we found that resveratrol-enhanced resistance was completely diminished in the *npr1–1* mutants (**Figure 2**). Moreover, resveratrol promoted nuclear accumulation of NPR1 (**Figure 3**), demonstrating that NPR1 is essential for resveratrol-mediated defense. In addition, nuclear accumulation of NPR1 depends on its monomerization and phosphorylation (Liu et al., 2005; Maier et al., 2011). Several kinases, including PKS5 and SnRK2.8, regulate these processes (Xie et al., 2010; Lee et al., 2015; Zhou et al., 2015; Backer et al., 2019). Because resveratrol promotes NPR1 nuclear accumulation, it likely to influence these regulatory mechanisms. Future studies should be performed to determine whether resveratrol directly or indirectly modulates NPR1-associated kinases or redox regulators to fine-tune NPR1 activation.

SA is major signaling molecule in plant immunity and functions through NPR1-dependent signaling cascade (Kim et al., 2022). Our data demonstrate that resveratrol treatment increased endogenous SA levels and upregulated the expression of key SA biosynthesis genes (**Figure 4**). Furthermore, resveratrol-induced resistance and *PR1* expression were abolished in *sid2* and *NahG* plants (**Figure 5**), which demonstrate that SA accumulation is required for resveratrol-triggered defense. Similar mechanisms have been reported for several naturally occurring compounds, including naringenin, kaempferol, quercetin, and rutin, which enhance resistance by promoting SA biosynthesis (Yang et al., 2016; An et al., 2021, 2022, 2023). Therefore, our findings support a model in which resveratrol promotes pathogen resistance through activation of the SA-NPR1 signaling module.

Interestingly, although SA accumulation was required for resveratrol-enhanced pathogen resistance, resveratrol-induced MPK activation occurred independently of SA (**Supplementary Figure 5B**). This result suggests that resveratrol activates multiple immune signaling branches. MPK activation likely occurs upstream of or parallel to SA biosynthesis, whereas SA signaling mediates downstream transcriptional reprogramming through NPR1. Further studies should be done to clarify the hierarchical relationship between MPK activation and SA biosynthesis and to determine how these pathways are coordinated to regulate immune responses.

### Resveratrol triggers ROS accumulation and redox-dependent immune amplification

ROS production represents a hallmark of plant immune responses (Jia et al., 2010). In agreement with this, our DAB staining assays showed that resveratrol markedly increased H_2_O_2_ accumulation in *Arabidopsis* leaves (**Figure 6**), indicating that ROS accumulation forms an integral component of the defense response activated by resveratrol. During plant immunity, apoplastic ROS are predominantly generated by NADPH oxidases, also referred to as respiratory burst oxidase homologs (RBOHs) (Torres et al., 2002). Although this study did not identify the specific ROS-producing enzymes, the substantial increase in H_2_O_2_ suggests that membrane-localized RBOHs contribute to this response.

Moreover, ROS influence cellular redox homeostasis and amplify defense signaling networks (Gahlowt et al., 2025). Importantly, ROS signaling is tightly linked with the SA signaling pathway (Torres et al., 2006). Alterations in intracellular redox environment facilitate the conversion of NPR1 oligomers into their active monomeric form, which enables NPR1 nuclear translocation and subsequent activation of defense gene expression (Mou et al., 2003). Therefore, resveratrol-induced ROS production may facilitate SA-dependent NPR1 activation by modifying cellular redox status. Based on these observations, we propose that ROS production and SA signaling act cooperatively to strengthen immune responses and enhance pathogen resistance. Future studies should be done to identify the specific RBOH isoforms and redox regulators involved and to determine how redox changes are integrated with SA biosynthesis and signaling.

### Proposed working model and future perspectives

Our findings support a model in which resveratrol enhances plant immunity through coordinated activation of SA- and ROS-dependent signaling pathways (**Figure 7**). Resveratrol increases the expression of genes involved in SA biosynthesis, resulting in increased endogenous SA contents. Increased SA facilitates the nuclear translocation and activation of NPR1, which in turn activates the expression of defense-related genes such as *PRs*. As a consequence, resistance to bacterial pathogens is enhanced. In parallel, resveratrol induces ROS production, which alters the cellular redox environment and strengthens immune signaling.

**Figure 7:**
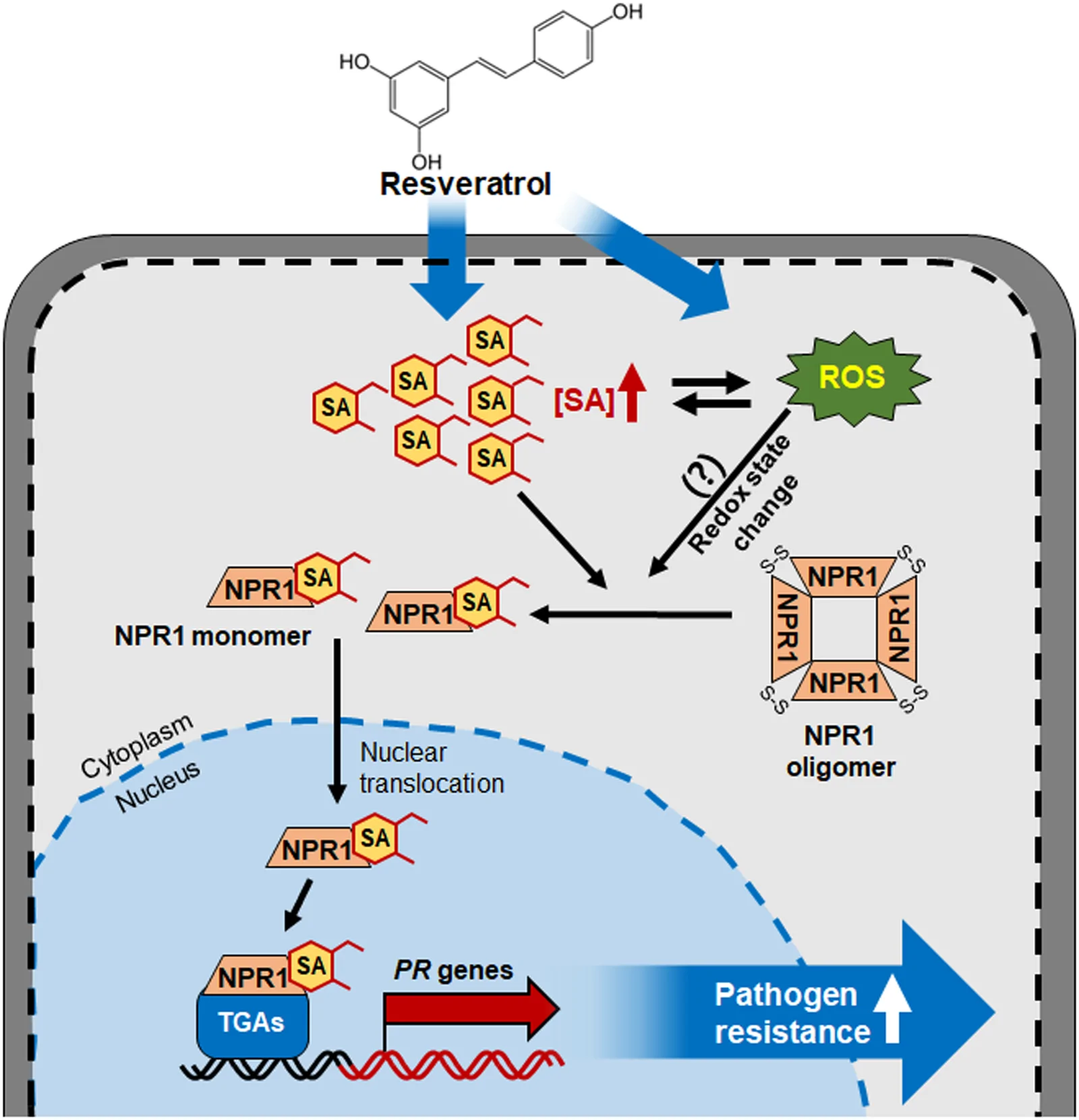
Proposed model showing resveratrol-induced pathogen resistance in *Arabidopsis*. Resveratrol increases the accumulation of intracellular SA and ROS. Elevated SA levels promote the conversion of NPR1 oligomer to monomer and induce NPR1 nuclear translocation. In the nucleus, NPR1 activates the transcription of *PR* genes through interaction with TGA transcription factors, thereby enhancing resistance to bacterial pathogens.

Despite these insights, several important questions remain. First, although our study demonstrates that exogenously applied resveratrol promotes plant immunity, it remains unclear whether endogenously synthesized resveratrol exerts similar functions in *Arabidopsis*, as previously proposed by Jeandet et al. (2013). Therefore, the conclusions of the present study are limited to the effects of exogenous resveratrol application and may not fully recapitulate the physiological effects of endogenously biosynthesized resveratrol. Further studies using resveratrol-producing transgenic plants will be necessary to determine whether endogenous resveratrol activates similar immune responses. Furthermore, the mechanism by which plants perceive resveratrol is currently unknown. It would be necessary to determine whether resveratrol is recognized by a specific extracellular or intracellular receptor and how resveratrol modulates immune signaling components. Lastly, the relationship between resveratrol-induced ROS production and SA biosynthesis should be clarified. Identification of resveratrol-responsive signaling components and their integration with established immune pathways would be essential to understand the action mode of resveratrol. A detailed mechanistic understanding of resveratrol signaling will clarify how this small molecule enhances plant immunity and will provide broader insight into the regulatory functions of plant secondary metabolites in defense networks.

## Data Availability

The original contributions presented in the study are included in the article/**Supplementary Material**; further inquiries can be directed to the corresponding author.
