# Supplementary material for: The resveratrol monomer enhances resistance to bacterial pathogen through the activation of SA-NPR1 signaling pathway in Arabidopsis
Source: Front Plant Sci. 2026 Sep 28;17:1834312. doi: 10.3389/fpls.2026.1834312 (PMC13655154; doi:10.3389/fpls.2026.1834312)
Supplement: Supplementary file 1 [file DataSheet1.docx]

**Supplementary Figure 1** Resveratrol does not exhibit antimicrobial activity against bacterial pathogens at concentrations up to 200 μM. (**A**) Antimicrobial activity was evaluated using a bacterial growth assay on a solid medium. *Pst* DC3000 was spotted onto KB medium supplemented with 0 to 200 μM resveratrol and incubated at 28℃. Photographs were taken 3 d after incubation. (**B**) Effect of resveratrol on the growth rate of *Pst* DC3000. Bacterial growth curves were generated in the KB liquid medium containing 0, 50, 100, 200, or 500 μM resveratrol. Optical density at 600 nm (OD_600_) was measured at 2 h intervals over a 24 h cultured period. Significant differences among resveratrol concentrations at each time point were analyzed by one-way ANOVA followed by Tukey’s test (***, P < 0.001; **, P < 0.01; *, P<0.05).

**Supplementary Figure 2** Expression patterns of *PR1* and *NPR* genes in response to resveratrol treatment. Total RNA was extracted from Col-0 plants treated with 100 µM resveratrol for the indicated time periods. Transcript levels of *PR1* (A), *NPR1* (B), *NPR3* (C), and *NPR4* (D) were quantified by RT-qPCR using gene-specific primers. *Tubulin* was used as an internal control. Bars represent the mean ± S.D. (*n* = 3). Different letters indicate statistically significant differences among time points, as determined by one-way ANOVA followed by Tukey’s test (P < 0.05).

**Supplementary Figure 3** Protein expression of PR1 and NPR1 after resveratrol treatment. Total proteins were extracted from Col-0 plant treated with 100 μM resveratrol for 0, 1, 3, 6, 12, and 24 h and separated by SDS-PAGE. PR1 and NPR1 proteins were detected by immunoblot analysis using anti-PR1 and anti-NPR1 antibodies, respectively. Rubisco bands stained with Coomassie Brilliant Blue (CBB) served as a loading control.

**Supplementary Figure 4** NPR3 and NPR4 do not affect resveratrol-induced resistance to bacterial pathogens. **(A)** Comparison of disease symptoms in Col-0, *npr3-2*, and *npr4-2* plants following resveratrol treatment. Plants were pretreated with mock or 100 μM resveratrol for 2 d and then inoculated with *Pst* DC3000 (5 × 10^5^ cfu/mL). Photographs were taken at 3 dpi. Scale bar = 0.5 cm. **(B)** Resveratrol-induced suppression of bacterial growth is not affected in the *npr3-2* and *npr4-2* mutants. Bacterial growth was determined by counting cfu at 3 dpi, as shown in (A). Each biological replicate consisted of three leaf discs collected from different leaves of the same plant. Bars represent the mean ± S.D. (*n* = 4). Different letters indicate statistically significant differences among genotype × treatment combinations, as determined by two-way ANOVA with Tukey’s post hoc test (P < 0.001).

**Supplementary Figure 5** Resveratrol activates MPK3 and MPK6. **(A)** Resveratrol induces MPK activation. Two-week-old Col-0, *mpk3*, and *mpk6* plants were treated with 100 μM resveratrol for the indicated time periods. Total protein extracts were prepared from these plants and separated by SDS-PAGE. MPK activation was detected by immunoblot analysis using anti-phospho-p44/42 MPK antibodies (α-p44/42, Cell Signaling Technology, USA). Rubisco bands stained with Coomassie Brilliant Blue (CBB) served as a loading control. Similar results were obtained in three independent experiments. **(B)** Resveratrol-induced activation of MPK is independent of SA. Two-week-old Col-0, *NahG* and *sid2* plants were treated with 100 μM resveratrol and analyzed as described in **(A)**. Similar results were obtained in three independent experiments.

**Supplementary Table S1. The primers used for RT-qPCR.**

| Genes | Position | Sequence |
| --- | --- | --- |
| Tubulin | F | 5'- *CCAACAACGTGAAATCGACAG* -3' |
|  | R | 5'- *TCTTGGTATTGCTGGTACTCT* -3' |
| PR1 | F | 5'- *GTGGGTTAGCGAGAAGGCTA* -3' |
|  | R | 5'- *ACTTTGGCACATCCGAGTCT* -3' |
| PR2 | F | 5'- *CGGTACATCAACGTTGGAA* -3' |
|  | R | 5'- *GCGTAGTCTAGATGGATGTT* -3' |
| PR5 | F | 5'- *CATGGATCAGAACAATGTCGT* -3' |
|  | R | 5'- *CTTGAAAATCCTCGAGTAGTC* -3' |
| SARD1 | F | 5'- *CCTCAACCAGCCCTACGTTA* -3' |
|  | R | 5'- *TAGTGGCTCGCAGCATATTG* -3' |
| EDS1 | F | 5'- *TCGAAGGGGACATAGATTGG* -3' |
|  | R | 5'- *CTTTTCATGTACGGCCCTGT* -3' |
| ICS1 | F | 5'- *AGTGAATTTGCAGTCAGTCGGGAT* -3' |
|  | R | 5'- *AATCGCCTGTAGAGATGTTGT* -3' |
| NPR1 | F | 5'- *AGTGAATTTGCAGTCAGTCGGGAT* -3' |
|  | R | 5'- *AGTGAATTTGCAGTCAGTCGGGAT* -3' |
| NPR3 | F | 5'- *ACGACATCCTCGACGATTTC* -3' |
|  | R | 5'- *GTGCAGGTCATCTCTTATGG* -3' |
| NPR4 | F | 5'- *GAAACCGTTCAACAACGCAG* -3' |
|  | R | 5'- *TAACGTCGTTCTTCAGCTCG* -3' |
